# Inflammation in the male genital tract: implications for HIV acquisition and transmission

**DOI:** 10.1186/1742-4690-9-S2-O23

**Published:** 2012-09-13

**Authors:** AJ Olivier, L Roberts, D Coetzee, A Williamson, JS Passmore, WA Burgers

**Affiliations:** 1 IIDMM, Division of Medical Virology, University of Cape Town, Cape Town, South Africa; 2 Department of Public Heatlh, University of Cape Town, Cape Town, South Africa

## Background

Elevated plasma levels of pro-inflammatory mediators such as TNFα, IL-1β, IL-6 and IL-8, MIP-1α, MIP-1β and RANTES have been demonstrated in HIV-infected individuals and HIV induces higher levels of pro-inflammatory cytokines in the female genital tract. We characterized levels of inflammation in semen, to gain an understanding of factors influencing transmission and acquisition in the male genital tract. Our hypothesis was that infected men would exhibit higher levels of inflammation in semen than uninfected men.

## Methods

We investigated concentrations of 20 pro-inflammatory and other mediators in the semen and blood of 38 HIV-infected and 42 uninfected men forming part of an HIV-discordant heterosexual couples study. We measured plasma and seminal viral loads to examine the relationship between viral replication and inflammation.

## Results

We found that the majority of cytokines/chemokines were at higher concentrations in semen than blood, both in HIV-infected and uninfected men. There were no significant differences between any cytokines/chemokines in the semen of HIV-infected vs uninfected men. We found that TNFα (p=0.013; r=0.55), G-CSF (p=0.0057; r=0.61), IL-10 (p=0.006; r=0.61) and IFNγ (p=0.01; r=0.57) seminal levels were significantly associated with increases in seminal viral load. Furthermore, subsequent to controlling for the effect of plasma viral load in a multivariate regression analysis, we found that both seminal IL-10 and IFNγ levels were associated with a significant rise in seminal viral load.

## Conclusion

Taken together, the data demonstrate that the immune milieu of the genital tract differs substantially from blood, with the majority of cytokines/chemokines tested elevated in semen. However, there were no differences in the levels of pro-inflammatory mediators in the semen of HIV-infected and uninfected men, or HIV-infected men on suppressive ART. Thus, even in the absence of HIV infection, the male genital tract appears to maintain a state of inflammation, which may have been the result of undetected and untreated co-infections.

